# Ontogeny, functions and reprogramming of Kupffer cells upon infectious disease

**DOI:** 10.3389/fimmu.2023.1238452

**Published:** 2023-08-25

**Authors:** Mohamed Amer Musrati, Patrick De Baetselier, Kiavash Movahedi, Jo A. Van Ginderachter

**Affiliations:** ^1^ Lab of Cellular and Molecular Immunology, Vrije Universiteit Brussel, Brussels, Belgium; ^2^ Myeloid Cell Immunology Lab, VIB Center for Inflammation Research, Brussels, Belgium; ^3^ Lab of Molecular and Cellular Therapy, Vrije Universiteit Brussel, Brussels, Belgium

**Keywords:** liver, Kupffer cell, liver macrophages, infectious diseases, macrophage ontogeny

## Abstract

The liver is a vital metabolic organ that also performs important immune-regulatory functions. In the context of infections, the liver represents a target site for various pathogens, while also having an outstanding capacity to filter the blood from pathogens and to contain infections. Pathogen scavenging by the liver is primarily performed by its large and heterogeneous macrophage population. The major liver-resident macrophage population is located within the hepatic microcirculation and is known as Kupffer cells (KCs). Although other minor macrophages reside in the liver as well, KCs remain the best characterized and are the best well-known hepatic macrophage population to be functionally involved in the clearance of infections. The response of KCs to pathogenic insults often governs the overall severity and outcome of infections on the host. Moreover, infections also impart long-lasting, and rarely studied changes to the KC pool. In this review, we discuss current knowledge on the biology and the various roles of liver macrophages during infections. In addition, we reflect on the potential of infection history to imprint long-lasting effects on macrophages, in particular liver macrophages.

## Liver macrophage heterogeneity and functions during homeostasis

### Ontogeny of Kupffer cells

**Figure 1:**
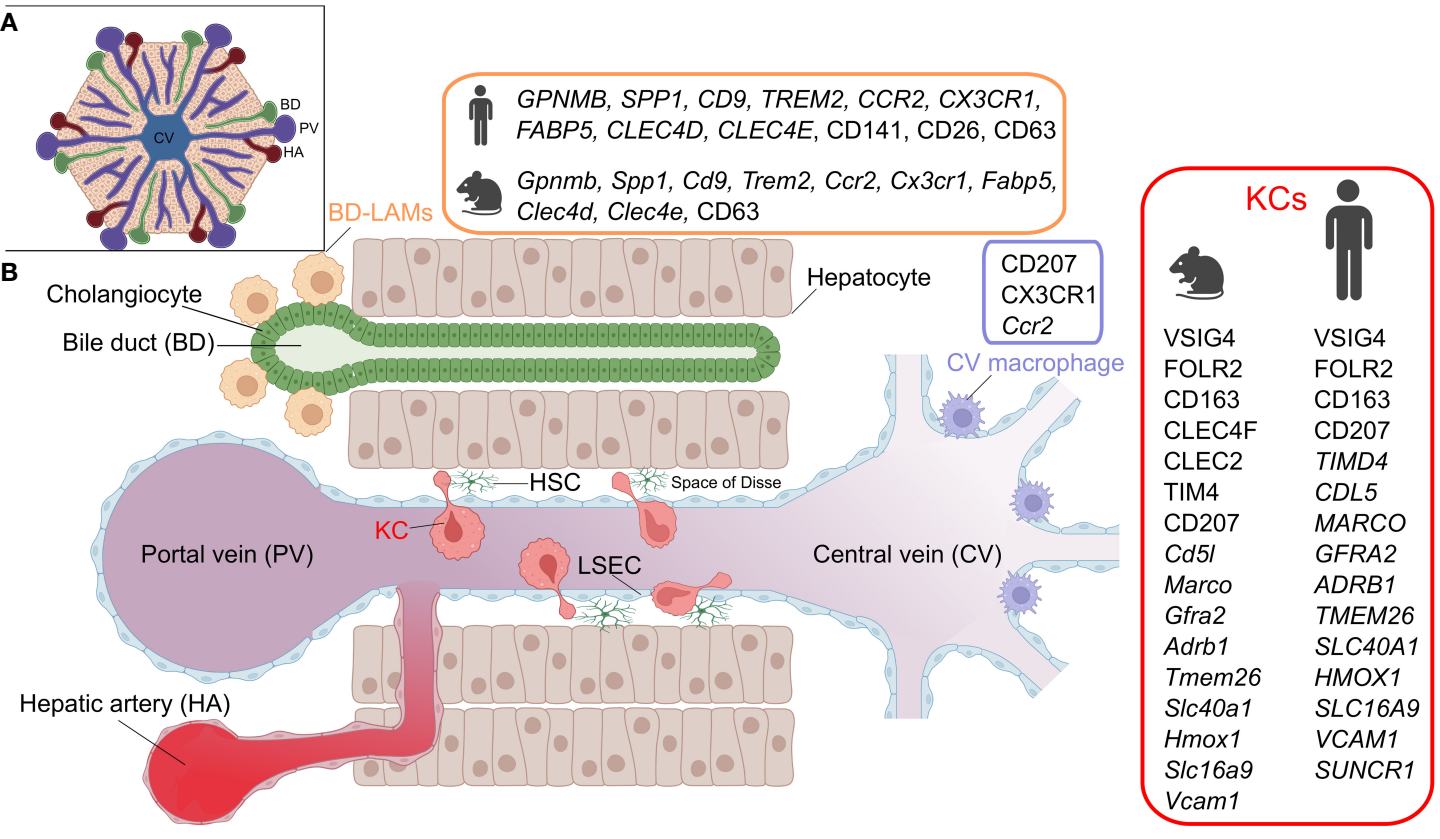
Hepatic macrophage subsets. A) The structure of the liver lobule. B) A cross section of liver sinusoids illustrating the different resident-macrophage populations. KC, Kupffer cell; LAM, lipid-associated macrophage; LSEC, liver sinusoidal endothelial cell; HSC, hepatic stellate cell. Marker details adapted from (1). Figure created with BioRender.com.

The liver harbors a large and heterogeneous population of resident macrophages (**Figure 1**). Work on mouse models has generated extensive information on the major resident macrophage population in the liver, known as KCs, which resides in the sinusoidal vessels and reaches into the perisinusoidal space of Disse towards hepatic stellate cells and hepatocytes (2). KCs are established embryonically and originate from yolk sac erythro-myeloid progenitors that seed the fetal liver by E10.5, later becoming heavily diluted by fetal liver monocytes (3–6), while also receiving a minor contribution from blood monocytes during the post-natal period (7). Studies have highlighted the importance of the transcription factors *Id3* and *Nr1h3* (coding for LXRα) for normal KC development and maintenance of identity, respectively (8, 9).

During homeostatic conditions, KCs self-maintain and are only marginally replenished by adult bone marrow-derived monocytes (3, 5, 10). However, as will be discussed in later sections, bone marrow contribution to KCs increases dramatically in response to certain liver insults that trigger KC depletion. An important question is whether ontogeny is important for KC functionality. Work utilizing a murine model of diphtheria toxin-mediated specific KC depletion (*Clec4f*-DTR mice), demonstrated that circulating monocytes engraft in the liver upon KC depletion and give rise to monocyte-derived KCs (moKCs) that are highly reminiscent of their embryonic KC (emKCs) predecessors, at least at the transcriptome level (7). Monocyte engraftment as moKCs is orchestrated by the cellular niche of KCs, comprised of hepatocytes, liver sinusoidal endothelial cells (LSECs), and hepatic stellate cells and mediated via niche-derived signals such as DLL4, bone morphogenetic proteins (BMPs), TGFβ and desmosterol (2, 11). In particular, the ALK1-BMP9/10 axis, involving ALK1 (KCs) and BMP9/10 (stellate cells), is of critical importance for KC homeostasis, as ALK1 loss leads to defects in KC development and maintenance (12, 13).

Phenotypically, murine KCs can be readily identified by their unique surface expression of the endocytic C-type lectin receptor CLEC4F (7, 14). Additionally, KCs can be distinguished from other liver macrophages by TIM4, CLEC1B and VSIG4 expression, although these markers can also be expressed by other tissue-resident macrophages outside the liver (14, 15). One outstanding current challenge is to phenotypically distinguish between moKCs and emKCs. Given that TIM4 is acquired in the late stages of KC development, several studies relied on the lack of TIM4 expression to define moKCs in models of non-alcoholic steatohepatitis and irradiation-induced liver injury (7, 16–18). However, it is likely that the temporal regulation of TIM4 acquisition is model-dependent. Indeed, in the context of natural KC development during embryogenesis, *Timd4* expression occurs relatively early and precedes the upregulation of other KC marker genes such as *Clec4f* (2). Likewise, *Cd163* mRNA has been shown to be mainly enriched in emKCs (7, 17). However, *in situ* translatome analysis using a Cre-driven RiboTag reporter revealed that emKCs and moKCs express similar levels of CD163 (19). One marker that appears to be highly enriched in emKCs, and lowly expressed by moKCs across models is MARCO (17, 19). However, whether MARCO expression by itself enables a clear distinction between the two subsets has not been tested. Consequently, genetic fate-mapping, using transgenic mouse strains that report bone marrow ontogeny, remains the most reliable and non-invasive method for separating em- and moKCs.

### Functions of Kupffer cells

KCs perform a wide array of homeostatic functions that support liver functionality and limit tissue injury. KCs play a key role in maintaining immunological **tolerance** in the liver under baseline conditions. For instance, human KCs secrete IL-10 in response to LPS stimulation *in vitro* (20). Murine KCs scavenge circulating particles, promote the expansion of Foxp3^+^IL-10^+^ T regulatory cells (21) and suppress dendritic cell-induced T-cell activation by the action of prostaglandins (22). Moreover, the liver is a major site for **iron metabolism** (23) and murine KCs are implicated in regulating iron homeostasis as they express key genes involved in macrophage heme-iron metabolism, such as *Spi-c* and *Nfe2l2* (24). Murine KCs scavenge damaged and aged red blood cells and are strategically located to deliver iron to hepatocytes (2, 24, 25). Murine KCs are also thought to control body iron levels by regulating hepcidin expression (26), although this remains controversial (27). In addition to their erythro-phagocytic capacity, murine KCs are implicated in **maintaining hemostasis** by contributing to the clearance of aged platelets (28, 29). This is mediated by the Ashwell-Morell receptor and macrophage galactose lectin, as well as CLEC4F (28, 29). As such, KC depletion in mice via clodronate liposomes was associated with adverse platelet dysfunctions, as manifested by increased bleeding following wounding (29). Furthermore, murine KCs participate in the efferocytosis of apoptotic cells (30), and are also thought to regulate circulating neutrophil numbers by mediating the clearance of apoptotic neutrophils (31, 32). KCs are also implicated in **lipid metabolism** as they are enriched in the expression of liver X-family of receptors (LXRs) and peroxisome proliferator-activated family of receptors (PPARs), involved in various aspects of cholesterol transport and metabolism (reviewed in (33)). Murine KCs are abundantly equipped with low density lipoprotein (LDL) receptor, and appear to serve as gatekeepers for LDL cholesterol transport to hepatocytes (34). In line with this, in *Csf1r* knockout rats, which exhibit major deficiencies in many tissue-resident macrophages including KCs, the liver shows a vast dysregulation of genes related to lipid metabolism, liver growth and function, and endures extensive age-progressive steatosis (35). Additionally, murine KCs have recently been shown to be the major source of miR-690 within the liver, a microRNA that negatively regulates hepatocyte lipogenesis and hepatic stellate cell fibrogenic activation (36). Deletion of miR-690 shifts KCs towards a pro-inflammatory polarization, suggesting that this microRNA regulates some KC functions in the steady state liver (36). Consistent with this, treatment with miR-690 can reverse the impaired erythro-phagocytic capacity of KCs associated with non-alcoholic steatohepatitis (36).

### Liver macrophage heterogeneity

A recent study has identified two distinct subpopulations of embryonically-derived KCs in mice: a major CD206^low^ESAM^–^ population (KC1) and a minor CD206^high^ESAM^+^ population (KC2) (37). Although they share the expression of core KC genes, KC1 mainly expresses genes associated with immune functions, while KC2 exhibits a higher expression of endothelial markers and genes associated with metabolic pathways such as lipid metabolism. Accordingly, the depletion of KC2 has been shown to prevent diet-induced obesity (37). Subsequent reports argued that the KC2 might represent endothelial cell-KC doublets, hence, these populations are still a current subject of debate (12, 38, 39).

Although KCs represent the major population of liver-resident macrophages, the steady state liver also hosts other macrophage populations that are spatially segregated (**Figure 1**) (12). These include bile duct-associated macrophages, which show transcriptional similarities to lipid-associated macrophages, expressing genes such as *Gpnmb*, *Spp1* and *Trem2* (12). Notably, a similar counterpart is also present in human livers (12). Moreover, another population of *Cd207*-expressing macrophages has been detected around the central veins of the murine liver (12). Apart from some data suggesting that murine bile duct macrophages exhibit a lower pro-inflammatory response to LPS stimulation than KCs, these newly identified macrophages generally lack functional characterization (12). Finally, the murine liver also harbors another population of macrophages residing in its capsule (40). These CX3CR1-expressing capsular macrophages are derived from monocytes, and accumulate after weaning in a microbiota-dependent manner and perform defensive functions against peritoneal pathogens (40).

### Human Kupffer cells

A number of studies sought to characterize KCs in human livers (1) and we hereby review notable findings. Single cell RNA-sequencing (scRNA-seq) of *CD163+VSIG4+* cells from human livers has revealed subpopulations with distinct gene expression signatures, including *MARCO*+*LILRB5*+ and *CD1C*+*FCER1A*+ populations, enriched in metabolic/immunoregulatory and antigen presentation pathways, respectively (41). Another study also reported a *VSIG4*+*MARCO*+ population based on scRNA-seq of human livers and has histologically shown that these cells are concentrated in periportal areas (42). Moreover, analysis of healthy and cirrhotic human livers identified two KC-like populations that were commonly enriched in *CD163*, *MARCO* and *CD5L,* while differing in *TIMD4* expression. Noteworthy, the *MARCO*+*TIMD4*- population was less present in the cirrhotic livers. Immunohistochemistry showed that while *TIMD4*+ cells were equally present in both the healthy and cirrhotic livers, *MARCO*+ cells were reduced during cirrhosis (43). A recent analysis of healthy human livers, using spatial proteo-genomics and single-cell sequencing approaches, identified a macrophage population that resembles murine KCs (12). The human KC-like population highly expressed core murine KC markers, including *CD5L, VSIG4, SLC1A3, CD163, FOLR2, TIMD4, MARCO, GFRA2, ADRB1, TMEM26, SLC4OA1, HMOX1, SLC16A9* and *VCAM1* (**Figure 1**), and appears to be localized in the mid zones of the liver lobules. Additionally, the same study detected a moKC-like hepatic macrophage subset in human livers, which expresses many of the core KC genes but lacks *TIMD4* expression (12). It is noteworthy that human KCs lack the expression of the specific murine KC marker CLEC4F, and in contrast to murine counterparts, express *SUCNR1* (12) (discrepancies in KC markers between species and studies were recently reviewed in (1)). Although delineating the ontogeny of KCs in humans is difficult to achieve, it is clear that humans generally endure a variety of pathological insults that affect the liver throughout life. Several insults that are known to alter KC ontogeny in mice, such as infections, a high-fat diet and alcohol consumption, are highly relevant for humans. It is therefore likely that the ontogeny of human KCs varies across individuals, and may possibly be a mixture of bone marrow-derived as well as embryonic KCs. Variability likely also exists at the level of the activation status of human KCs since exposure to inflammatory episodes can imprint prolonged changes on macrophages, as elaborated later in this review.

## Liver macrophages in the context of infections

### Bacterial infections

The liver has an outstanding capacity to filter circulating bacteria (44, 45). This superior microbial scavenging property is underlined by the presence of KCs in the hepatic vasculature (44, 46). Due to their remarkable pattern of zonation around the periportal tracts of the liver blood vessels (47), KCs are among the first cells to encounter the incoming antigen-rich portal blood. KCs form an extensive scavenger network that actively monitors sinusoidal blood and captures circulating bacteria (48–50). KCs are equipped with a large repertoire of phagocytic receptors that facilitates the clearance of bacterial pathogens (49–51). A well characterized KC receptor that plays an essential role in bacterial clearance is VSIG4 (48–50). In addition to serving as a complement receptor for C3-opsonized bacteria (50), VSIG4 can directly bind the lipoteichoic acid component of the gram-positive bacterial cell wall, enabling their phagocytosis by KCs (48). Indeed, loss of *Vsig4* results in enhanced bacterial dissemination and increased mortality in mice infected with *Listeria monocytogenes* (50). Deletion of *Vsig4* in a mouse model of alcoholic liver disease hinders the phagocytosis of *Enterococcus faecalis* by KCs, and associates with increased systemic dissemination of gut-translocated bacteria as well as exacerbated pathology (52). Noteworthy, decreased *Vsig4* expression and a reduced percentage of VSIG4^+^ macrophages is seen in the livers of patients with alcohol-related liver disease (52). Aside from the direct role in bacterial clearance via phagocytosis, KCs can indirectly instruct other innate immune cells to launch anti-microbial responses. For example, during *Borrelia burgdorferi* infection, murine KCs present antigen to invariant natural killer T cells (iNKT) in a CD1d-dependent manner, leading to iNKT cell activation and interferon-γ production (46). Activated iNKT cells in turn restrict the extra-hepatic dissemination of bacteria (46). Furthermore, the capture of certain bacterial pathogens such as *Bacillus cereus* and Methicillin-resistant *Staphylococcus aureus* (MRSA) by murine KCs has been shown to induce platelet aggregation on KCs (53). This process requires interactions between GPIb and GPIIb on platelets with von Willebrand factor (vWF) on KCs. Platelet aggregation appears to serve an important protective function in response to *B. cereus* infection in mice as GPIbα deficiency leads to increased mortality, impaired bacterial clearance, exacerbated liver damage and vascular injury (53). Interestingly, murine KCs possess dual-track mechanisms to remove bacteria that permit either the slow or fast clearance of bacterial pathogens (49). The former clearance route is regulated by time-dependent opsonization of bacteria by platelets via interaction with complement proteins. These bacteria-platelet complexes exhibit a relatively increased half-life in the circulation and are subsequently captured by VSIG4 on KCs. Conversely, the fast clearance route is mediated by scavenger receptors in a platelet-independent manner, hence allowing the rapid removal of bacteria (49). This dual-track clearance mechanism ensures the balance between rapid removal of bacteria while also maintaining antigen availability for the priming of adaptive immune responses (49). Interestingly, the anti-bacterial properties of murine KCs are strongly regulated by the gut microbiome (particularly the bacterial microbiome), and depletion of the gut microbiota associates with defective KC responses and results in higher vulnerability to infection (47, 54). Remarkably, the spatial distribution of KCs around the periportal tracts in the liver is in itself microbiota-dependent (47). Mechanistically, microbiome-derived signals regulate the composition of the extracellular matrix of LSECs in a MyD88-dependent manner, causing CXCL9 retention on periportal LSECs, hence allowing interaction with CXCR3 on KCs (47). Moreover, continuous crosstalk with gut commensals can directly shape the bacterial killing properties of KCs in a microbiome-derived, D-lactate-dependent manner (54). In addition, murine KCs exhibit a loss of their typical phenotype, gene signature and spatial zonation upon the deletion of *Alk1*, and this associates with impaired bacterial clearance and ultimately increased mortality in response to bacterial infection (13).

Intriguingly, encounter with some bacterial pathogens seems to compromise KC viability, resulting in the death and transient depletion of KCs. For instance, the facultative intracellular bacterial pathogens *L. monocytogenes*, as well as *Salmonella enterica* serovar Typhimurium, are known to infect murine liver macrophages leading to macrophage death (55). KCs respond to *L. monocytogenes* infection by undergoing necroptosis (55), a form of programmed lytic cell death via Receptor-Interacting Protein Kinase 3 (RIPK3) activation of Mixed Lineage Kinase Domain-like (MLKL) (56). Dying KCs become massively replaced by CCR2-dependent, monocyte-derived macrophages that proliferate intrahepatically, driven by signals including CSF1, hepatocyte-derived IL-33 and basophil-derived IL-4 (55). Interestingly, *L. monocytogenes*-induced KC necroptosis is thought to serve a beneficial host response by orchestrating a type 2 inflammation that limits excessive liver injury. Mechanistically, the necroptosis of KCs promotes hepatocyte production of IL-33, which presumably induces IL-4 production by basophils and thereby the alternative activation of liver macrophages (55). Paradoxically, another study reported that *L. monocytogenes* induces rapid cell death in murine liver macrophages in an IRF3-dependent manner, and showed that *Irf3* deletion rescues KC death and reduces hepatic bacterial burden (57), suggesting a pathogenic consequence associated with KC death in this context. Notably, no restoration of emKCs is observed following *L. monocytogenes* infection, and instead, recruited moKCs appear to dominate and engraft durably in the liver after the clearance of infection (55). Whether this change alters KC responses to subsequent bacterial infections is unknown. However, murine moKCs have been shown to exhibit a markedly superior phagocytosis of a number of bacterial pathogens, including *L. monocytogenes* (17), suggesting that the modulation of KC ontogeny might lead to functional consequences. In contrast, *in vitro* examination has shown that moKCs generated in the *Clec4f*-DTR mouse model are equally phagocytic as emKCs (7). Nevertheless, the latter finding might reflect functional changes associated with the loss of the natural KC microenvironment, or even the artificial, non-physiological method of moKCs generation. Noteworthy, the phagocytic capacity of KCs can be altered by exposure to environmental factors. For instance, certain clinical drugs have been shown to negatively modulate the scavenging properties of murine KCs (58, 59). For example, tacrolimus, a commonly used drug to prevent graft rejection after solid organ transplantation and to treat graft-versus-host disease after bone marrow transplantation, has been shown to inhibit bacterial uptake and killing by KCs in mice (59). In humans, the risk of *Staphylococcus aureus* infection is associated with higher tacrolimus concentrations in liver transplant recipients (59). An overdose of acetaminophen has also been shown to imprint an immune-suppressive profile on murine KCs by upregulating PD1 at the cell surface, and to impair their phagocytic properties (58). Intriguingly, contrasting the negative effect of the aforementioned drugs on KCs, physical exercise has been shown to boost the phagocytic capacity of murine KCs, and to increase their expression of the phagocytic receptors MARCO and SR-A, which associates with enhanced endotoxin clearance from the blood circulation (60). Moreover, exercise training imprints a long-lasting anti-inflammatory profile on murine KCs, manifested in a lowered production of pro-inflammatory cytokines and an enhanced secretion of IL-10 and IL-1Ra in response to an LPS challenge (61). This exercise-induced anti-inflammatory reprogramming of KCs is regulated by itaconate metabolism, and can be recapitulated by KC treatment with itaconate, whereas the deletion of *Irg1* (which codes for cis-aconitate decarboxylase) abrogates this beneficial effect of exercise in mice (61). Importantly, the livers of exercised mice undergo less injury and necrosis following an LPS challenge (61), thus highlighting the effects of physical exercise in modulating the response to infectious inflammation in the liver. Aside from the various roles played by KCs during microbial infections, murine liver capsular macrophages are also implicated in the response to bacteria. They are thought to limit peritoneal pathogen dissemination to the liver by recruiting neutrophils, although the underlying mechanism remains vague (40).

In summary, in addition to their typical scavenging role, KCs regulate complex processes that facilitate the containment of bacterial infections and tissue injury. Moreover, the bactericidal properties of KCs are subject to regulation by environmental factors.

### Viral infections

Hepato-tropic viruses, such as hepatitis B virus (HBV), can establish life-long persistent infections (62) and KCs seem to be implicated in the different aspects of HBV pathogenesis. Mouse experiments utilizing clodronate liposome-mediated KC depletion have suggested that KCs exhibit suppressive effects on CD8^+^ T cells during HBV infection (63). As such, KC depletion lessens HBV persistence and associates with enhanced numbers and activation of CD8^+^ T cells (63). Interestingly, KC-mediated suppression of CD8^+^ T cells has been shown to be TLR2-dependent, as KCs from WT but not TLR2 KO mice abundantly produced the CD8^+^ T cell-suppressive cytokine IL10 following *in vitro* stimulation with the hepatitis B core antigen (63). Murine KCs have also been reported to use an IL-10-dependent mechanism to promote systemic specific humoral tolerance to HBV (64). Recent work further explored the cellular and molecular mechanisms responsible for CD8^+^ T cell dysfunction during HBV infection. To dissect the consequences of hepatocellular-mediated priming versus KC-mediated priming of CD8^+^ T cells, a recent study performed cell-selective HBV targeting to either hepatocytes (natural target of HBV) or KCs (non-natural targets of HBV) in mice. This approach revealed that hepatocellular priming of virus-specific CD8^+^ T cells generates dysfunctional CD8^+^ T cells with impaired antiviral capacity (65). Conversely, KC-mediated priming leads to CD8^+^ T-cell differentiation into effector cells endowed with potent antiviral functions (65). KC-primed CD8^+^ T cells exhibit higher expression of activation markers such as *Gzma*, *Gzmb* and *Ifng* and show increased expression of *Il2*, the critical T-cell growth factor (65, 66). In line with these observations, treatment with recombinant IL-2 (IL-2-c) rescues and restores the antiviral functions of hepatocellularly-primed murine CD8^+^ T cells (65). Follow-up work has shown that the ability of IL-2-c treatment to rejuvenate dysfunctional CD8^+^ T cells during HBV infection is mediated by the KC2 subset, that is enriched in the expression of IL-2 signaling components (67). As such, the depletion of KC2 significantly impairs the efficacy of IL-2-c treatment (67).

Viral infections are also known to elicit dynamic compositional changes in the liver macrophage pool (68, 69). For instance, rapid IRF3-dependent KC death is seen in mice following infection with human adenovirus (57). Intriguingly, KC death during these settings appears functionally relevant. Indeed, although KC death was spared in IRF3 knockout mice, these mice exhibited higher hepatic viral loads. These data suggested either a pathogenic role for KCs during adenovirus infection, or an IRF3-mediated macrophage death pathway that serves a protective role during this infection (57). KC death also occurs after intravenous delivery of adenovirus vectors in mice (70). This was shown to be complement C3-dependent and to require VSIG4 expression, given that KC death is rescued in VSIG4- or C3-deficient mice (71). The process of KC death and replenishment by monocytes has also been explored in the context of vaccinia virus (VACV)-induced acute hepatitis in mice (69). During VACV infection, IFNAR triggering in myeloid cells, including KCs, contributes to the early control of the infection (69). In these settings, KCs are depleted and become replenished by infiltrating monocytes that differentiate into moKCs. Interestingly, bone marrow chimera experiments revealed that IFNAR signaling in monocytes delays their differentiation into moKCs (69). Hence, IFNAR-knockout monocytes engrafted more efficiently in the livers of VACV-infected mice than WT monocytes. This appeared to be a result of a direct IFNAR triggering of monocytes in the liver, as both WT and IFNAR knockout moKCs became equally engrafted in the KC population upon cessation of hepatic IFNAR triggering (69).

In conclusion, KCs clearly perform various and occasionally contrasting roles during viral infections.

### Parasitic and fungal infections

KCs are implicated in the pathology of several parasitic infections, and their contribution ranges from beneficial to detrimental. For instance, during experimental trypanosome infections, KCs perform an essential role in parasite clearance, as KC depletion by clodronate liposomes results in uncontrolled parasitemia and rapid mortality (72). In fact, VSIG4 is critical for the anti-trypanosomal response of KCs as it contributes to parasite clearance in a complement C3-dependent manner (72). Furthermore, murine hepatic macrophages, including KCs, are thought to be a source of CXCL16 during *Trypanosoma brucei* infection, and are hence suspected to be implicated in the recruitment of pathogenic CXCR6^+^ CD4^+^ T cells that promote aggravated liver damage and increased mortality during infection (73). Noteworthy, VSIG4-complement interactions seem of crucial importance to universal KC defense responses, since KCs utilize a similar mechanism to contain fungal infections (74). This is highlighted by impaired blood clearance and heightened infection burden in clodronate liposome-treated, as well as VSIG4 knockout or C3 knockout mice following *Cryptococcus neoformans* infection (74).

Remarkably, some parasites manage to circumvent the clearance by KCs. In case of *Plasmodium* infection, sporozoites rapidly target the liver to establish hepatocyte infection (75). Strikingly, experimental work has shown that sporozoites traverse through KCs to cross the endothelial barrier and infect hepatocytes (76–78). It has been illustrated that sporozoite crossing of KCs induces extensive KC death, with only a small fraction of KCs appearing to survive the crossing event (78). Although sporozoites can also traverse through LSECs, this process appears less common than KC traversal (78). Consistent with this, *in vitro* work showed that LSECs are less permissive to sporozoite traversal relative to KCs (76), overall suggesting that sporozoites utilize and favor KC crossing to overcome the sinusoidal lining and establish hepatocyte infection. Furthermore, it appears that sporozoites modulate KC functionalities to favor their intracellular survival, as illustrated by *in vitro* work showing that sporozoites can suppress the respiratory burst in KCs by increasing intracellular cAMP (79). This mechanism is mediated by the parasite circumsporozoite protein, and is inhibited by the blockade of CD91 or the removal of surface proteoglycans on KCs (79). By altering intracellular levels of cAMP, sporozoites are also thought to inhibit inflammatory cytokine production by KCs, instead favoring IL-10 secretion (80). Additionally, the phagocytic capacity of murine KCs towards bacteria has been shown to be substantially impaired during *Plasmodium* infection (81), further highlighting the compromising effects of malarial infection on KCs. The notion that KCs might exhibit pathogenic features during malarial infection is further supported by the finding that osteopetrotic (op/op) mice, which show a severe deficiency of mononuclear phagocytes including KCs, exhibit reduced hepatic infection (82). Paradoxically, depletion of KCs by clodronate liposomes augments parasitemia and hepatic infection (78, 82). Notably, clodronate liposome-mediated depletion of KCs is thought to compromise the structural integrity of the hepatic sinusoids, particularly by inducing gaps in the sinusoidal wall (82). Although this has been speculated to be the reason for the contradiction between the infection outcome following the aforementioned methods of KC-depletion, the authors provide no data to indicate that the integrity of the sinusoidal lining is unaltered in op/op mice (82).

Moreover, in a murine model of self-resolving *Plasmodium* infection invoked by the injection of parasitized erythrocytes, KCs are partially lost and replaced by infiltrating monocytes that in turn gave rise to long-lasting moKCs (83). In this model, KC loss occurs prior to the peak of blood parasitemia and coincides with an increase in the infiltration of monocytic cells (83). Notably, *in vitro* work has also suggested a role for ferroptosis and the deposition of hemozoin (81), a crystallized by-product of hemoglobin catabolism that is released in the circulation during malarial infection (84), as a trigger of KC death during *Plasmodium* infection (81). It is worth mentioning that a common feature in the pathology of many parasitic infections, including those invoked by *Plasmodium* and *Trypanosoma*, is the occurrence of anemia. In such settings, erythrocytes exhibit surface alterations resulting in enhanced erythro-phagocytosis by phagocytic cells (85, 86). As they are implicated in the clearance of damaged erythrocytes (24, 25), the erythro-phagocytic functions of KCs might also contribute to their response to parasitic infections. In this regard, erythro-phagocytosis has been shown to imprint an anti-inflammatory signature on KCs that is manifested in a lower expression of *Ifng*, *Tnf*, *Il12b*, *Cxcl9* and *Cxcl10* and MHCII-related genes in a mouse model of hemolytic anemia (87). Whether reprogramming due to erythro-phagocytosis functionally contributes to the response to parasitic infections is unclear.

During schistosomiasis, the main pathology results from the host type 2 immune response to the parasite eggs deposited in the liver. The eggs become surrounded by a granulomatous reaction encompassing mononuclear cells, eosinophils, neutrophils and fibroblasts, ultimately resulting in fibrosis (88). Alternatively activated macrophages, including KCs and monocyte-derived macrophages, are implicated in mediating the type 2 immune response and pathology in this context. Murine *Schistosoma mansoni* infection leads to the accumulation of Ly6C^hi^ monocytes in the liver, which proliferate and differentiate into CD11b^hi^ macrophages that gradually become the predominant hepatic macrophage population during the infection (89). KCs are steadily ablated as the infection proceeds, and although they showed little, if any, proliferative activity, they remain mainly of embryonic origin up to 10 weeks post-infection. Nevertheless, at this time-point, a minor but significant bone marrow contribution to the KC pool was observed (89). CD11b^hi^ macrophages are alternatively activated in the infected livers and exhibit an IL4Ra-dependent upregulation of Ym1 and Relm-α. Although granuloma size was increased upon the deletion of *Il4ra* in myeloid cells (*Lyz2^Cre^*), this did not alter the survival kinetics (89). A recent scRNA-seq of *Schistosoma japonica*-infected murine livers also provided insights into macrophage heterogeneity during schistosomiasis (90). This study reported the presence of two major macrophage populations in the infected liver, a KC population (*Timd4*, *Cd163*, *Marco*), and a *Ccr2*+ population that lacked the expression of KC markers, and might hence represent infiltrating monocyte-derived macrophages (90). Noteworthy, the two populations were enriched in *Chil3* expression, although KCs were notably more enriched in *Arg1* and *Retnla,* suggesting a general alternative activation of macrophages in this context (90). Moreover, hepatic macrophages from *S. mansoni*-infected mice produce IL-6, IL-10, IL-4 and IL-13 in response to stimulation with soluble worm antigens and are thought to present worm antigens to hepatic T cells, promoting their type 2 differentiation (91). KCs are also implicated in tapeworm infection, particularly in cystic echinococcosis, as they uptake, via CLEC4F, the mucins of the laminated layer that protect the larval stages (hydatids) of *Echinococcus granulosus* parasites (92).

Overall, while KCs mount protective responses in some types of parasitic infections, they seem to be overwhelmed and even hijacked in other cases of parasitic insults. Notably, most experimental work investigating the roles of KCs in parasitic infection models has been done using non-specific KC targeting and identification methods that complicates the interpretation of the results. Hence, the recent advances in KC targeting and identification techniques might help resolve ambiguous aspects of KC biology during parasitic infections.

## Perspectives: infection history and reprogramming of Kupffer cell function

As detailed previously, KCs are highly implicated in the response to pathogens. Given the wide range of pathogens that directly or indirectly target the liver, KC-pathogen interactions are probably a recurrent phenomenon during infections. In this regard, an increasing number of studies have shown that exposure to infections can alter tissue macrophage properties in the long-term. This effect can be mediated by ontogenic shifts, transcriptional and epigenetic reprogramming as well as alteration of the macrophage microenvironment (93–95). It is therefore likely that KCs are similarly subject to such modulation and that their functional properties are continuously shaped by infection history. For instance, although monocytes replenish KCs during almost all types of infectious diseases, it is unknown whether moKCs generated under these inflammatory scenarios are functionally distinct from emKCs. Although several studies report that moKCs generated following artificial KC-depletion during steady-state adopt the emKC identity and share some functional properties (7, 11, 17), this might not necessarily apply to situations where moKCs are generated in an inflammatory context. In support of this notion, it has been shown that monocyte-derived alveolar macrophages (moAMs), generated during influenza infection, are transcriptionally and epigenetically distinct from their embryonic counterparts and exhibit superior antibacterial functions (94). However, these enhanced antibacterial properties fail to develop when moAMs are generated following clodronate liposome-induced depletion of alveolar macrophages (94), confirming the need of inflammation to confer distinctive properties on monocyte-derived macrophages. In line with this, studies have highlighted some functional differences between emKCs and the moKCs generated during inflammatory contexts (17, 96). For instance, irradiation-generated moKCs are clearly superior to emKCs in the phagocytosis of a number of bacterial pathogens *in vivo* (17). In contrast, moKCs generated under lipemic conditions appear inferior to emKCs in their capacity to load and process lipids (96). Likewise, emKCs are more efficient in scavenging acetylated low density lipoprotein than irradiation-generated moKCs (17), indicating differences in the context of lipid metabolism. Moreover, emKCs are relatively radio-resistant as they upregulate the expression of *Cdkna1* to resist and survive irradiation, whereas moKCs seem to lack this property (19). Importantly, although some of the distinctions between moKCs and emKCs might readily be made on the basis of the transcriptome, some may be represented in forms of “hidden” alterations at the level of the epigenome. In this context, despite several weeks of intrahepatic residency after irradiation, moKCs only recover less than half of the tissue-specific enhancer regions found in emKCs (14), further supporting the idea that ontogeny or the timing of moKC generation associates with differential functionality.

In addition to ontogeny-related effects, pathogens may directly alter macrophages, or their microenvironment. For instance, exposure to pathogens or their components is well documented to drive a persistent remodelling of the epigenomic landscapes of macrophages (95, 97). For instance, peripheral LPS administration can reprogram murine microglia and associates with altered responses to neurological disease (97). Likewise, exposure to inflammatory signals commonly encountered during infections, such as interferon-γ (paradigm of type 1 inflammation) or IL-4 (paradigm of type 2 inflammation), reshapes the macrophage epigenome and triggers the generation of latent enhancers associated with faster and more robust responses upon secondary stimulation (98, 99). Although this concept is rarely studied in KCs, a recent study has shown that *in vitro* treatment of KCs with β-glucan induces trained immunity, promoting an anti-inflammatory response to a secondary stimulation with LPS (61), confirming that KCs are amenable to reprogramming. Tissue macrophages, including KCs, rely on local signals provided by stromal cells in their tissue of residence in order to maintain their identity (2, 100–103). It is therefore logical that changes to the KC niche as a result of infections might subsequently reprogram KC identity and functions. This concept is exemplified in the lung, where changes in the microenvironment resulting from a previous exposure to experimental pneumonia can epigenetically reprogram embryonically-derived alveolar macrophages, leading to long-term impairment of their phagocytic function (95). In conclusion, despite the fact that KCs frequently and dynamically respond to infections, studies exploring whether a past exposure to infectious inflammation alters the KC phenotype are largely lacking. Major questions include whether infection history alters KC biology, and if so, for how long that lasts, and whether such effects are reversible. Importantly, what is the physiological consequence of such changes on the overall response to secondary inflammation? Given the frequent exposure of humans to infections and the important roles played by liver macrophages during infections, this topic warrants further investigation.

## Author contributions

The manuscript was written by MM and revised by PB, KM and JG. All authors contributed to the article and approved the submitted version.
